# A New Method for Detecting Myocardial Ischemia Based on ECG T-Wave Area Curve (TWAC)

**DOI:** 10.3389/fphys.2021.660232

**Published:** 2021-03-31

**Authors:** Ronghua Li, Xiaoye Zhao, Yinglan Gong, Jucheng Zhang, Ruiqing Dong, Ling Xia

**Affiliations:** ^1^Key Laboratory for Biomedical Engineering of Ministry of Education, Institute of Biomedical Engineering, Zhejiang University, Hangzhou, China; ^2^Department of Medical Imaging Technology, North Minzu University, Yinchuan, China; ^3^Department of Clinical Engineering, 2nd Affiliated Hospital, School of Medicine, Zhejiang University, Hangzhou, China; ^4^Dushu Lake Hospital Affiliated to Soochow University, Suzhou, China

**Keywords:** myocardial ischemia, ECG, coronary heart disease, heart, electrophysiology

## Abstract

In recent years, coronary heart disease (CHD) has become one of the main diseases that endanger human health, with a high mortality and disability rate. Myocardial ischemia (MI) is the main symptom in the development of CHD. Continuous and severe myocardial ischemia will lead to myocardial infarction. The clinical manifestations of MI are mainly the changes of ST-T segment of ECG, that is, ST segment and T wave. Nearly one third of patients with CHD, however, has no obvious ECG changes. In this paper, a new method for detecting MI based on the T-wave area curve (TWAC) was proposed. Through observation and analysis of clinical data, it was found that there exist significant correlation between the morphology of TWAC and MI. The TWAC morphology of normal subject is smooth and gentle, while the TWAC morphology of patients with coronary stenosis is mostly jagged, and the curve becomes more severe with more severe stenosis. The preliminary test results show that the sensitivity, specificity, and accuracy of the proposed method for detecting MI are 84.3, 83.6, and 84%, respectively. This study shows that the TWAC based approach may be an effective method for detecting MI, especially for the CHD patients with no obvious ECG changes.

## Introduction

Cardiovascular disease has long been the first cause of death. In China only, the current number of patients with cardiovascular disease is 290 million, among them 11 million are patients with coronary heart disease (CHD) (Ma et al., 2020). For patients with typical CHD, diagnosis can be made based on resting electrocardiogram (ECG), exercise ECG, cardiac ultrasound, coronary angiography, and cardiac magnetic resonance. The resting ECG is widely used in clinic due to its non-invasive, economical and simple reasons. ST-T wave change is the most commonly used ECG feature to judge myocardial ischemia (MI) (Lin and Guo, 2005). According to related research, the positive rate of resting ECG diagnosis for CHD is 71%, and the rest of 29% patients have no ECG changes and some patients with three lesions can be completely normal, indicating that patients with normal ECG cannot rule out CHD (Pijls et al., 1995).

The clinical manifestations of MI are mainly the ST-T segment changes of the electrocardiogram, that is, the ST segment and T wave. The diagnosis of MI by ECG mainly depends on the characteristics of the ST-T segment. However, there are many factors affecting ST segment changes, such as axis shift, heart rate, electrode effects, body position changes, etc., leading to inaccurate detection of feature points, relying on ST segment shift to detect MI has a large false detection rate and missed detection rate. T wave represents the repolarization process of the ventricles. During MI, myocardial repolarization is delayed, resulting in changes in T wave morphology, such as biphasic T wave, inverted T wave, or high-tip T wave. In view of this characteristic of T wave, this paper proposes a method for diagnosing myocardial ischemia based on the T-wave area curve. By locating T-wave characteristic points, calculating T-wave area and drawing a curve with cardiac cycles, based on clinical data, the qualitative relationship between myocardial ischemia and T-wave area curve was analyzed.

## Materials and Methods

### T-Wave Onset and Offset Detection

T wave is an important part of ECG signal. Accurate localization and morphological recognition of T wave are basic indicators for diagnosis of MI, but the shape of T wave is variable. Hayden et al. (2002) show that when ischemic cardiomyopathy occurs, the T-wave shape will change accordingly, such as inverted, bi-phase, high-point, etc., and the low-frequency components near the end of the T-wave are more abundant than other bands, and more susceptible to noise and baseline drift. Accurate finding the location of the T-wave end point is with some difficulties. Therefore, T-wave detection needs to take into account the changes in T-wave morphology.

At present, T-wave ends can be detected by methods such as area method (Zhang et al., 2006; Vázquez-Seisdedos et al., 2011), wavelet transform (Martínez et al., 2004), pattern recognition (Saini et al., 2013), and artificial neural network (Maglaveras et al., 1998). Due to the noise of the T wave and the baseline drift, the wavelet transform method may contain aliasing part. Therefore, simply using the wavelet method has a higher false detection rate, and some waveforms caused by noise or baseline drift are falsely detected as T waves. The neural network method can adapt to the change of T-wave shape when it is used for feature detection. It has good robustness, but the algorithm is more complicated. For the area-dependent methods, the algorithms can adapt to abnormal changes in T-wave shape, and have strong waveform adaptabilities. Among them, Zhang’s algorithm (Zhang et al., 2006) is a good method for T-wave ends location. It was based on an indicator signal with mathematically proved consistency. It was robust to measurement noise, waveform shape changes and baseline drift, and is suitable for various forms of T waves. The computation burden of the algorithm was very low: its main computation can be implemented as a simple FIR filter. When evaluated with the PhysioNet QT database (Goldberger et al., 2000) in terms of the mean and the standard deviation of the T-wave end location errors, Zhang’s algorithm outperforms the other algorithms evaluated with the same database (Zhang et al., 2006). So, in this study, Zhang’s algorithm is basically used for T-wave ends location.

**FIGURE 1:**
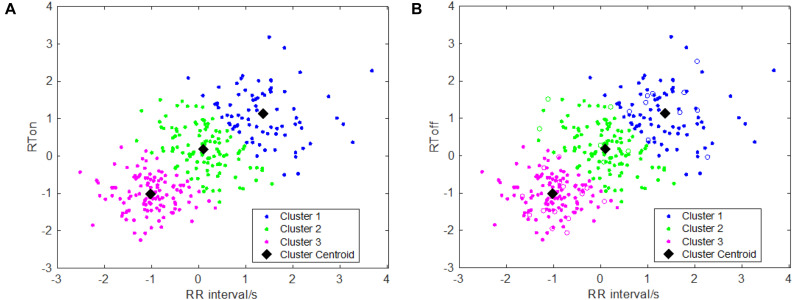
Clustering results for T wave feature points: **(A)** clustering information of T wave onsets; **(B)** clustering information of T wave offsets.

One key issue of the area-dependent methods is to accurately determine the search boundaries, but the search boundaries are closely related to the RR interval. If the interval of the searching window’s boundaries was set too small which means that two boundary points are near the current *R* peak, the maximum of sliding area could not be found or the detected onset/offset of *T* wave are nearer to the *R* peak. This issue will affect detection accuracy, which results in detection error and vice versa. In order to more accurately model the relationships between RR interval and the searching boundaries, in this study, similar to Shang et al.’s work (Shang et al., 2019), we performed a k-means clustering analysis between RR intervals and R*T*_*on*_ (R*T*_*on*_ denotes the time interval between the R peak and T wave onset) as well as the relationship between the RR intervals and R*T*_*off*_ (R*T*_*off*_ the time interval between the R peak and T wave offset), which is implemented by means of the k-means function. The scatter plots with the optimal k-means clustering (*k* = 3) are showed in Figure 1, and k is determined by combining the results of clustering and the computational complexity of parameters’ settings as well as the adaptiveness of the algorithm. Then, the two relationships (between RR intervals and R*T*_*on*_, and between RR intervals and RT_*o*__*ff*_) are obtained using the following equations:

(1) $$\begin{array}{c} C\,a\,s\,e\,1:R\,R<0.67\,s,0.05\,s<R\,T_{o\,n}<0.25\,s\\ Case2:0.67s\leq RR<1.23s,0.05s<RT_{o\,n}<0.35s\\ C\,a\,s\,e\,3:R\,R\geq 1.23\,s,0.05\,s<R\,T_{o\,n}<0.45\,s\\ C\,a\,s\,e\,1:R\,R<0.71\,s,0.2\,s<R\,T_{o\,f\,f}<0.45\,s\\ Case2:0.71s\leq RR<1.1s,0.2s<RT_{o\,f\,f}<0.6s\\ C\,a\,s\,e\,3:R\,R\geq 1.1\,s,0.2\,s<R\,T_{o\,f\,f}<0.45\,s \end{array}$$

Thus, the three piecewise functions for determining the search boundaries for *T* wave onset and offset detections can be obtained.

Then, the grid search was used to determine the best combination of parameters in Equations (2) and (3), which was implemented by for loop. In a loop, we changed the value of one parameter at a time, kept the other parameters unchanged, and applied the algorithm in the QT database as well as using a fivefold cross-validation. Then, we stored the sensitivity of one loop and started another loop. Through all loops, we traversed all of the combinations of parameters. After comparing the results, the combinations of parameters with the highest sensitivity were chosen. The best parameters’ combinations for *T* wave onsets are: a = 0.4, b = 0.2, c = 0.4, d = 0.4, e = 0.3, f = 0.0, and for T wave offsets are: a = 0.2, b = 0.1, c = 0.2, d = 0.0, e = 0.0, f = 0.1.

(2) $$\left\{ \begin{array}{c} t_{1}=\left(\left\lceil a\times \sqrt{R\,R_{i}}\right\rceil +R_{i}+0.02\right)\,s\\ t_{2}=\left(\left\lceil b\times \left(R\,R_{i}\right)\right\rceil +R_{i}+0.16\right)\,s\,\text{if}\,R\,R_{i}<0.67\,s\\ t_{1}=\left(\left\lfloor c\times \sqrt{R\,R_{i}}\right\rfloor +R_{i}+0.04\right)\,s\\ t_{2=}\,\left(\left\lfloor d\times \left(R\,R_{i}\right)\right\rfloor +R_{i}+0.24\right)\,s\,i\,f\,0.67\,s\leq R\,R_{i}<1.23\,s\\ t_{1}=\left(\left\lceil e\times \sqrt{R\,R_{i}}\right\rceil +R_{i}+0.04\right)\,s\\ t_{2}=\left(\left\lceil f\times \left(R\,R_{i}\right)\right\rceil +R_{i}+0.4\right)\,s\,\text{if}\,R\,R_{i}\geq 1.23\,s \end{array}\right.$$

(3) $$\left\{ \begin{array}{c} t_{1}=\left(\left\lceil a\times \sqrt{R\,R_{i}}\right\rceil +R_{i}+0.18\right)\,s\\ t_{2}=\left(\left\lceil b\times \left(R\,R_{i}\right)\right\rceil +R_{i}+0.3\right)\,s\,\text{if}\,R\,R_{i}<0.71\,s\\ t_{1}=\left(\left\lfloor c\times \sqrt{R\,R_{i}}\right\rfloor +R_{i}+0.18\right)\,s\\ t_{2=}\,\left(\left\lfloor d\times \left(R\,R_{i}\right)\right\rfloor +R_{i}+0.4\right)\,s\,\text{if}\,0.71\,s\leq R\,R_{i}<1.1\,s\\ t_{1}=\left(\left\lceil e\times \sqrt{R\,R_{i}}\right\rceil +R_{i}+0.18\right)\,s\\ t_{2}=\left(\left\lceil f\times \left(R\,R_{i}\right)\right\rceil +R_{i}+0.48\right)\,s\,\text{if}\,R\,R_{i}\geq 1.1\,s \end{array}\right.$$

### T-Wave Area Curve

When the T-wave onset and offset was detected, the T-wave area can be calculated as follows.

Let the T wave onset be *T*_*on*_ and the T wave offset be *T*_*off*_. Within the fixed window t ∈ [*T*_*on*_, *T*_*off*_], calculate the waveform area *A_t*:

(4) $$A_{t}=\sum_{t=T_{o\,n}}^{T_{o\,f\,f}}\left(s_{t}-\bar{s_{k}}\right)$$

where *s_t* is the amplitude of the t-th sample point, and $\bar{s_{k}}$ is the local average amplitude (by default, a smoothing window of *p* = 0.016 s is used), which is defined as:

(5) $${\bar{s}}_{k}=\frac{1}{2\,p+1}\,\sum_{j=t-p}^{t\,p}S_{j}$$

Calculate the continuous T wave area of each lead, and then draw the connection line with the cardiac cycle number as the abscissa and the T wave area as the ordinate. Based on the T wave morphological variability during MI, it can be inferred that if the line is approximately straight, the ischemia test is negative. If one or more of the leads are serrated, it is positive, and if one or more of the leads are serrated, it may be related to the position of the blocked coronary artery is related.

From the T-wave area curves of 52 healthy samples in the PTB database, it was found that the curves of 43 healthy people were flat and the morphological differences were small. The difference in T-wave area per heartbeat of patients with MI, however, is obviously larger than that of healthy people. The area curve is jagged and irregular. Observing the corresponding T-wave area curve of the 15-s electrocardiogram data of 148 patients with MI in the PTB database, it was found that the curves of 115 MI patients were irregular and chaotic, which was in sharp contrast with the curves of healthy samples.

According to the characteristic that the degree of fluctuation of the T-wave area curve of patients with MI is significantly greater than that of healthy people, we used the TWAC (T-wave area curve) to detect MI. Based on the TWAC form, the gentle TWAC is defined as negative, corresponding to myocardial blood supply was normal in the subject. And the irregular jagged TWAC was defined as positive, corresponding to myocardial ischemia in the subject.

### Experimental Data

The sample number in this article is 364 in two groups: the first group contains 148 patients with MI and 52 normal persons from the PTB database (Goldberger et al., 2000); the second group contains 122 patients with suspected MI from Zhejiang Second Hospital and 42 health subjects. The detailed characteristics of the selection patients are shown in Table 1.

**TABLE 1:** Clinical characteristics of patients.

**Characteristics**	**Myocardial ischemia**		***p*-value**
	**Yes (*n* = 236)**	**No (*n* = 128)**	
Age	59 ± 10	55 ± 9	0.025*
Female	106/236 (45%)	49/128 (39%)	0.163
Chest pain	130/236 (55%)	51/128 (40%)	0.023^[*dollar*]^
Dyspnea	94/236 (40%)	55/128 (43%)	0.58
Heart rate (bpm)	71 ± 8	70 ± 7	0.239
Ejection fraction (%)	65 ± 6	66 ± 2	0.012*
Left ventricular end diastolic diameter (mmHg) (mm)	47 ± 4	46 ± 2	< 0.01*
Systolic blood pressure (mmHg)	132 ± 27	122 ± 10	0.303
Diastolic blood pressure (mmHg)	76 ± 9	74 ± 6	0.03*
Smoke	113/236 (48%)	55/128 (43%)	0.5
Hypertension	144/236 (61%)	68/128 (53%)	0.197
Diabetes mellitus	73/236 (31%)	33/128 (26%)	0.354
Dyslipidemia	170/236 (72%)	78/128 (61%)	0.032^[*dollar*]^
Family history of CAD	42/236 (18%)	18/128 (14%)	0.396

P-value are obtained using independent-samples t-test and Chi-square test. * Significant differences between groups using independent-samples t-test. ^$^Significant differences between groups using Chi-square test. Diagnostic criteria for hypertension: systolic blood pressure ≥140 mmHg in three resting days on the same day; or diastolic blood pressure ≥90 mmHg; or those with a history of hypertension who are taking antihypertensive drugs and are currently at normal blood pressure. Diabetes diagnosis criteria: fasting blood glucose > 7.0 mmol/L or 2 h after meals > 11.1 mmol/L; or patients with a history of diabetes who are taking drugs to treat the current normal blood sugar. Diagnostic criteria for hyperlipidemia: total blood cholesterol > 6.00 mmol/L, or triglycerides ≥ 1.69 mmol/L during this hospitalization; or those with a history of hyperlipidemia who are currently taking hypolipidemic drugs and have normal blood lipids. Smoking history is based on past history.

The clinical data of Zhejiang Second Hospital was conducted from May 2018 to December 2018. Philips TC20 ECG machine was used to record resting ECG data with a sampling rate of 1,000 Hz and 16-bit resolution. We select stable 15 s ECG data (15 s ECG data contains about 20 heart beats) to draw a 12-lead T-wave area curve. This study was approved by Zhejiang Second Hospital Review Board. After signing the informed consent, the patient was placed in the supine position, and the resting ECG was obtained after strictly following the 12-lead ECG collection procedure. Each patient was subjected to coronary angiography after ECG examination. A professional cardiology interventional doctor performed a visual assessment of the degree of coronary artery stenosis. In order to ensure the accuracy of the ECG results of this study, a professional electrocardiologist interpreted the ECG data, but he did not know the coronary angiography examination results of the patients.

Selection criteria were patients with suspected MI who had no MI characteristics on the ECG. The absence of MI characteristics means that the ST segment of any lead with R wave as the main wave is not depressed or depressed < 0.05 mv, T wave is upright and ≥ 1/10R wave. Suspicious MI refers to the clinical manifestations, myocardial enzymes and other tests and coronary CTA and other examinations suggest that the patient may have MI, which is evaluated by a professional cardiologist.

Exclusion criteria were non-sinus electrocardiograms such as atrial fibrillation and atrial flutter; premature beats; atrioventricular block or ventricular block; ventricular pre-excitation patterns; abnormal Q waves or poor R-wave increments in right chest leads; significant sinus Bradycardia (heart rate < 50 beats/min); heart valve disease.

This article used the results of CAG or blood flow reserve fraction (FFR) as the gold standard for MI diagnosis, and defined coronary angiography result as a positive MI if one of the three branches of the coronary artery (anterior descending branch, circumflex branch, right coronary artery) has a stenosis degree of ≥ 70% (Tonino et al., 2009); all without stenosis or a degree of stenosis less than 70% are negative MI. Patients are considered positive MI when FFR value ≥ 0.8, and negative MI if FFR value < 0.8.

## Results

**FIGURE 2:**
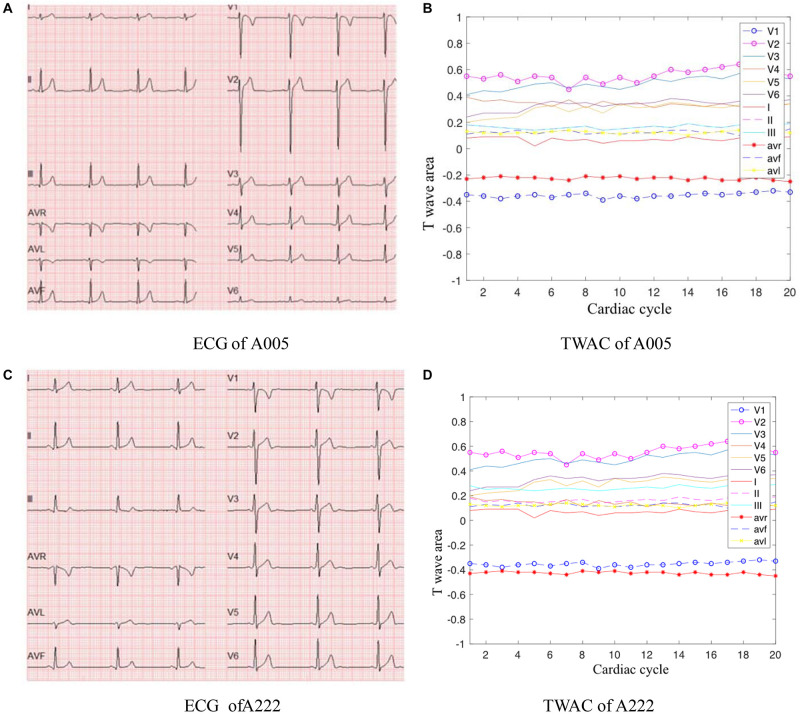
ECG and TWAC Examples of two normal subjects: A005, male, 29 years old; A222, male, 23 years old. **(A)** ECG of A005; **(B)** TWAC of A005; **(C)** ECG of A222; **(D)** TWAC of A222.

**FIGURE 3:**
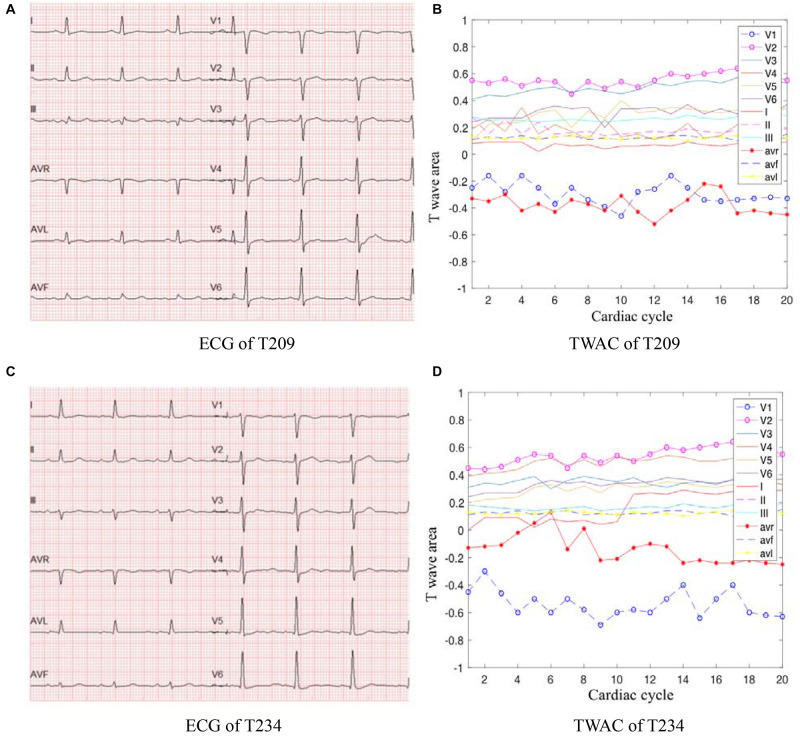
ECG and TWAC examples of two MI patients: T209, female, 66 years of age, with moderate coronary stenosis; T234, male, 73 years, with severe coronary stenosis. **(A)** ECG of T209; **(B)** TWAC of T20; **(C)** ECG of T234; **(D)** TWAC of T234.

As mentioned above, TWAC is a curve of the T-wave area of a conventional 12-lead ECG as a function of the cardiac cycle. By analyzing the TWAC morphology of a lot of clinical data, it is found that the curve of normal people has less fluctuation, and the curve of patients with MI is mostly jagged changes in different cardiac cycles. Figure 2 shows an example of TWAC in two normal people, and Figure 3 shows an example of TWAC in two patients with MI. It shows that the T-wave area curve has a significant correlation with ischemic heart disease. Therefore, in this article, TWAC of one or more leads with jagged fluctuations is identified as positive (with MI), and the fluctuations are small and gentle in TWAC is considered as negative (no MI).

Experiment results are shown in Table 2. The sensitivity of TWAC to the diagnosis of MI was 84.3% (199/236), the specificity was 83.6% (107/128), and the accuracy was 84.0% (306/364).

**TABLE 2:** Experimental results, where TN, TP, FN, and FP represent true negative, true positive, false negative, and false positive, respectively.

**Experiment samples**	**TWAC**		**TP, TN, FP, FN**				**SEN (%)**	**SPE (%)**	**ACC (%)**
	**Positive**	**Negative**	**TN**	**TP**	**FN**	**FP**			
PTB 148 MI	119	29	–	119	29		80.4	–	80.4
PTB 52 healthy	9	43	43	–	–	9	–	82.7	82.7
ZJU 122 MI	89	33	25	80	8	9	89.9	75.8	86.1
ZJU 42 healthy	3	39	39	–	–	3	–	92.9	92.9
Total 364	220	144	107	199	37	21	84.3	83.6	84.0

*The sensitivity (SEN), specificity (SPE) and accuracy (ACC) are defined as follows:*

$Sensitivity:\overset{\text{def}}{=}\frac{T\,P}{T\,P+F\,N}\left(\%\right)$

$Specificity:\overset{\text{def}}{=}\frac{T\,N}{T\,N+F\,P}\left(\%\right)$

$Accuracy:\overset{\text{def}}{=}\frac{T\,P+T\,N}{T\,P+T\,N+F\,P+F\,N}\left(\%\right)$

## Discussion

### Analysis of Misjudgment Data

The total number of patients in the false-negative group was 37, of which 13 patients had a diseased vessel stenosis of 70%. The method used in this study to interpret the degree of coronary stenosis was physician visual assessment (PVA), which is based on the personal experience of the surgeon. Judgment is relatively subjective. Studies have pointed out that the severity of stenosis of coronary lesions in PVA in China is significantly higher than quantitative coronary angiography (QCA) and there is a large difference between hospitals and doctors (Zhang et al., 2018). Therefore, the presence of false-negative patients does not rule out the possibility of overestimation of coronary artery disease caused by PVA. Single-vessel disease is more common in false-negative patients, while multiple-vessel disease is more common in true-positive patients. The range of MI caused by single vessel disease is relatively small, and negative results are easily obtained. In addition, collateral circulation is another factor of false negative results. The collateral circulation rate of patients in the false negative group is higher than that in the true positive group. Professional cardiologists have confirmed that there are 4 cases of coronary stenosis >70% in the false negative group. Collateral circulation can improve the heart blood supply and may make TWAC negative.

A total of 21 false-positive patients was included in this study, 15 of whom had chest tightness and chest pain. Although coronary angiography showed negative results, the possibility of MI could not be completely ruled out. In the FAME study, 35% of the lesions with a degree of coronary stenosis of 50–70% had an FFR of < 0.8, and in Park’s study, 16% of the lesions with a degree of coronary stenosis of < 50% had an FFR of < 0.8. The data indicate that MI is still possible without significant coronary stenosis (Tonino et al., 2009; Park et al., 2012; Ahmadi et al., 2015). The clinical manifestations of all these false positive patients were typical CHD and showed a higher incidence of coronary atherosclerosis than the true negative group.

### Analysis of the Relationship Between TWAC Morphology and Coronary Occlusion in Specific Leads

When the TWAC is positive, by observing the TWAC morphology on each lead, we’ve found that in addition to the *V*_*1*_,*V*_*3*_,*V*_*4*_ leads have obvious sharp points or inverted phenomena, the aVL lead and aVR lead curves also fluctuate greatly, as shown in Figure 3D.

T wave can reflect the heterogeneity of ventricular repolarization in patients and predict cardiovascular disease to a certain extent. T wave is a potential wave formed by repolarization of ventricular cells. Repolarization is an active energy-consuming process. When MI occurs, the heart cannot normally deliver blood and nutrients. Therefore, insufficient supply of myocardial energy will cause myocardial contraction and diastolic function is impaired, which may cause T wave changes in patients with ischemic cardiomyopathy.

Studies have shown that the degree of T wave changes in aVL leads reflects the degree of ventricular muscle excitement recovery time, and reflects the heterogeneity of ventricular muscle repolarization. Compared with ST-T changes, aVL lead T-wave changes have a higher sensitivity for the diagnosis of myocardial ischemia, which is significantly related to the number of coronary artery disease vessels and the degree of myocardial ischemic injury (Tepetam et al., 2016).

Studies have also shown that the inversion of T waves on leads *V*_3_ and *V_4* may indicate a middle obstruction of the left anterior descending coronary artery, which indicates that changes in T waves on specific leads can predict not only extensive myocardial ischemia, but also blockage at specific coronary arteries (De Zwaan et al., 1982). From the azimuth point, leads *V*_3_ and *V_4* correspond to the proximal anterior descending branch, and the coronary direction of the middle part of the left anterior descending branch is almost parallel to the aVL lead of the ECG. It can be speculated that if the adjacent area of the left anterior descending branch ischemia, the T wave of lead aVL should also change. Therefore, the T wave change of lead aVL also corresponds to the anterior descending branch of the left anterior descending coronary artery.

As shown in Figure 3B, the aVR lead curve fluctuates in a zigzag manner, and the coordinate points in some cardiac cycles exceed the abscissa and become positive values. In a normal 12-lead ECG, the T wave on the aVR lead is inverted. This patient’s CAG showed 70% stenosis of the left circumflex branch. Studies have shown that the morphological changes of the T wave in lead aVR are of great significance in predicting cardiovascular death, and its value is higher than other ECG leads, comparable to the changes in the ST segment of the aVR lead (Tan et al., 2008). If the amplitude of the inverted T wave becomes smaller, it means that the risk of cardiovascular death is gradually increased. When the inverted T wave becomes upright, the risk of cardiovascular death is higher.

The aVR lead has been used only to judge the origin of the heart rhythm, and its role has been seriously underestimated. The aVR lead has a special position on the frontal six-axis system, that is, the aVR lead axis is between the I and II lead axes, the angle between the aVR lead axis and the ventricular depolarization vector is the smallest, and the projection is the largest, which is the most sensitive lead to the changes of the ventricular depolarization vector.

### Advantages and Limitations

Coronary atherosclerosis causes a series of electrophysiological changes that affect ventricular repolarization (Downar et al., 1977; Janse and Wit, 1989). During cardiac ischemia, the duration of action potential and conduction velocity decrease, leading to a heterogeneous repolarization process (Janse et al., 1985). Studies have shown that ischemia increases the repolarization dispersion between normal and ischemic fibers, and between the epicardium and the endocardium (Coronel et al., 1988), which refers to the “every other heartbeat” on the ECG. The repolarization pattern has a continuous fluctuation (Arini et al., 2014). This fluctuation refers to the change in the amplitude of the T wave or the change in the ST segment between different cardiac cycles. The amplitude of these bipolar alternations (dispersions) is usually in the microvolt range and cannot be visually recognized. Computer-based signal processing and analysis technology can detect subtle ECG changes. The T wave amplitude and shape on ECG alternately change from beat to beat, which is called T wave electrical alternation (TWA) (Puletti et al., 1980). TWA represents the alternation of cardiac repolarization, is an indicator of ventricular tachycardia and ventricular fibrillation in ischemic myocardium, and can be used as an indicator of risk stratification of acute myocardial infarction. The disadvantage of TWA is that it is susceptible to breathing, electrode and skin interference, wire movement and body position changes, and further research is needed. TWAC analyzes subtle ECG signal changes and amplifies such subtle changes to detect abnormal dynamics of cardiac repolarization, and is robust to acquisition noise, baseline drift and T wave morphology.

TWAC is the T-wave area curve of 12 leads with the cardiac cycle. Different leads of the electrocardiogram record electrical signals at different positions of the heart. Therefore, the degree of fluctuation of TWAC on different leads reflects the degree of stenosis at specific blood vessels to a certain extent. This paper analyzed the TWAC and coronary angiography results of some patients, and found that the T wave changes on leads V_3_,V_4_, aVL corresponded to the obstruction of the left anterior descending branch of the coronary artery, and the T wave changes on the aVR lead predicted the stenosis at left trunk coronary arteries.

There are some limitations in this study. First, coronary angiography shows that coronary artery stenosis is not equivalent to MI (Tonino et al., 2009; Park et al., 2012; Ahmadi et al., 2015). Clinically, it can be directly intervened when the diameter of coronary artery is narrower than 90%; if the diameter of coronary artery is not narrower than 90%, it is recommended that only FFR ≤ 0.8, or the disease with corresponding evidence of ischemia, then intervention can be taken. For moderate coronary stenosis, even the experienced cardiologist’s interventional physician’s visual assessment of angiography cannot accurately evaluate its physiological significance (Fischer et al., 2002). The number of patients with mismatched TWAC and CAG results in this study was 58, of which 33 (56.7%) patients had coronary stenosis with a coronary stenosis of 40–70%. It cannot be ruled out that coronary angiography could not determine the myocardial ischemia relatively accurately. The accuracy rate of FFR value ≤ 0.80 for identifying myocardial ischemia caused by coronary artery stenosis is as high as >90%. It has been confirmed by extensive randomized controlled studies that FFR is the gold standard for evaluating the physiological significance of coronary artery stenosis. Changes in hemodynamic factors such as contractile force. In future studies, FFR will be considered as the gold standard for diagnosis of MI, which can further verify the accuracy of TWAC. Secondly, the sample size is small, and more clinical research is needed to further verify the TWAC method.

## Conclusion

In this paper, a new method for detecting MI was proposed, unlike the conventional ST-T segment based approaches, it was based on the T-wave area curve (TWAC). Preliminary test results show that the proposed method has good sensitivity, specificity, and accuracy for MI detection, especially for the CHD patients with no obvious ECG changes.

## Data Availability Statement

The raw data supporting the conclusions of this article will be made available by the authors, without undue reservation.

## Ethics Statement

The studies involving human participants were reviewed and approved by the Zhejiang Second Hospital Review Board. The patients/participants provided their written informed consent to participate in this study.

## Author Contributions

RL and XZ contributed to the algorithm and the statistical analysis. JZ and RD contributed to the clinical data collection and interpretation. YG, RD, and LX contributed to the funding acquisition, conception, and design of the study. All authors contributed to the writing, critical reading, and approval of the manuscript.

## Conflict of Interest

The authors declare that the research was conducted in the absence of any commercial or financial relationships that could be construed as a potential conflict of interest.
